# *Bupleuri Radix* Prevents the Recurrences of Resected Colonic Polyps by Affecting Angiogenin-2-Induced Protein Kinase B/Akt Signaling

**DOI:** 10.1155/2020/3531652

**Published:** 2020-10-28

**Authors:** Qiang Gao, Guihong Yu, Minghui Yu, Xinping Wang

**Affiliations:** ^1^Department of Spleen and Stomach Disease, Yantai Hospital of Traditional Chinese Medicine, Yantai 264002, China; ^2^Department of Integrated TCM & Western Medicine, Yantai Qishan Hospital, Yantai 264000, China; ^3^Department of Clinical Laboratory, Yantai Wanhua Hospital, Yantai 260000, China; ^4^Department of Clinical Laboratory, Yantai Hospital of Traditional Chinese Medicine, Yantai 264002, China

## Abstract

**Aim:**

We aimed to explore the effects of Bupleuri Radix (BR) on the recurrence of resected colonic polyp (CP) by measuring angiogenin-2-induced protein kinase B (Ang PKB)/Akt signaling.

**Method:**

The main ingredients of BR were extracted by using ethanol and measured by HPLC. One hundred twenty patients with CP >10 mm who underwent resected surgery were randomly allocated to an aspirin (AG) or a BR medicine (BG) group. The allocation ratio was 1 : 1 and the intervention duration was one year. The recurrence rate of resected CP was investigated and the plasma levels of Ang PKB/Akt and inflammatory cytokines were measured using ELISA kits. After one-year surgery, side effects were recorded. The relationship between the serum levels of the main compounds of BR and plasma levels of Ang PKB/Akt was analyzed.

**Results:**

The main ingredients of CP are paeoniflorin, baicalin, saikosaponin A, and bupleurum saponin B2. Recurrence of resected CP was found in 17 patients from the AG group and eight patients from the BG group after one-year follow-up (*p* < 0.05). The levels of angiogenin-2 II and PKB/Akt in the AG group were higher than those in the BG group (*p* < 0.05). Meanwhile, BR treatment reduced the plasma levels of TNF-*α*, IL-1*β,* and IL-6, and increased the level of IL-10(*p* < 0.05). Inflammatory cytokines are important factors that affect the recurrence of resected CP. Serum paeoniflorin, baicalin, saikosaponin A, and bupleurum saponin B2 in BR had a strong negative relationship with the plasma levels of Ang PKB/Akt.

**Conclusion:**

BR significantly reduces the recurrence risk of resected CP by affecting Ang PKB/Akt signaling.

## 1. Introduction

A colonic polyp (CP) is a polyp growing on the surface of the colon, and untreated CP is the more likely precursor to develop colorectal cancer in most patients [1]. No effective drug has been proven or is recommended to treat CP yet. Long-term use of nonsteroidal anti-inflammatory drugs (NSAIDs), notably aspirin, may reduce the rates of cancerous CP [2] and colon cancer [3] and rectal cancer [4]. However, the side effects of aspirin are also remarkable, such as rapid breathing and seizure; severe nausea, vomiting, and stomach pain; vomit and fever [5]. Therefore, it is necessary to explore natural products with few side effects for prevention of CP risk.

*Bupleuri Radix* (BR) is a famous traditional Chinese herb and exhibits various biological activities such as anticancer, antimicrobial, and antiviral properties [6, 7]. Paeoniflorin, naringin, sennoside A, baicalin, baicalein, and saikosaponin A are the main components of BR [8] and have been reported to show inhibitory function on various cancers [9–11]. However, its function on CP development remains unclear. Angiogenesis will promote polyp growth and progression [12]. BR is one of main herbs having antiangiogenic effects [13]. Angiogenin-2-induced protein kinase B (Ang PKB)/Akt signaling is necessary for angiogenesis [14]. Thus, BR possibly inhibits CP progression via Ang PKB/Akt signaling. We aimed to explore the effects of BR on the recurrence of resected CP by investigating the expression of Ang PKB/Akt and the relationship between serum main components of BR and plasma Ang PKB/Akt.

## 2. Materials and Methods

### 2.1. HPLC Analysis of BR Extracts

BR roots were purchased from Yantai Zhengsu Medical Technology Co., Ltd. (Yantai, Shandong, China). Dried and pulverized roots (10 g) of BR were extracted with 95% ethanol (1 L, 1 h), and the extracts were evaporated in vacuum to produce dark-brown residues. The residues were resuspended in 95% ethanol to a final volume of 10 mg/mL and filtered using a MF-Millipore membrane filter (0.45 *µ*m pore size) and then directly applied to an Agilent 1200 series HPLC system (Santa Clara, CA, USA). The HPLC analysis was carried out with an Agilent TC-C18 column (250 mm × 4.6 mm, 5 *μ*m), the mobile phase acetonitrile (A)-0.1% phosphoric acid aqueous solution (B), gradient elution (0∼10 min, 5%∼15% A; 10∼50 min, 15%∼42% A; 50∼60 min, 42%∼68% A), column temperature 30°C, flow rate 1.0 mL/min, detection wavelengths between 230 nm, and injection volume 10 *μ*L. Meanwhile, paeoniflorin (lot number: PRF9031342, purity: 98.91%), baicalin (lot number: PRF9091201, purity: 98.68%), saikosaponin A (lot number: PRF9022503, purity: 98.18%), and bupleurum saponin B2 (batch number: RF9062242, purity: 99.79%) were purchased from Chengdu Prefa Technology Development Co., Ltd. (Chengdu, China). All these standards were measured under the same HPLC condition.

### 2.2. Participants

This study was approved by the Ethics Committee of Yantai Hospital of Traditional Chinese Medicine and performed according to the Helsinki Declaration. All patients and controls provided their written informed consent. From March 3, 2017, to July 3, 2018, 298 patients had CP diameter > 10  mm and would receive endoscopic resection of CPs at Yantai Hospital of Traditional Chinese Medicine, and all data would be analyzed by our authors.

### 2.3. Inclusion Criteria

The following inclusion criteria were used: (1) ≥40 years of age; (2) an endoscopic CP >10 mm in diameter; (3) complete medical records.

### 2.4. Exclusion Criteria

The following were the exclusion criteria: abnormal intestinal position due to congenital malformations or surgery; a thicker than normal abdominal wall; absence of polyps in patients; polyps being either unresected or observed, but not retrieved; patients who had a history of colectomy or proctectomy.

### 2.5. Sample Size Calculation

Sample size was calculated based on 95% confidence interval (*α* = 5%) and 80% power (*β*  = 10%) and a sampling formula [15]: *n* = (2 × (*Zα* + Z*β*)^2^ × SD^2^)/*d*^2^, where *Zα* = 1.96, *Zβ* = 1.28 [16]. The size of CP was the main variable of interest, 22.5 was the SD of CP size percent detected in the previous experiment, and 10% was postulated as a clinically significant difference in CP size between the two groups (d). Therefore, *n* = (2 × (1.96 + 1.28)^2^ × 22.5^2^)/10^2^ = 106.6. Considering 10% possibility of dropouts, *n* = 60 in each group were recruited.

### 2.6. Endoscopic Resection of CPs

CPs were resected by specially trained endoscopists using snare polypectomy or biopsy forceps. The method for endoscopic resection was chosen based on a surgery procedure. A circumferential incision was conducted using an electrosurgical knife (Spectronics, Westbury, NY, USA), and then submucosal dissection was performed. Medical results were recorded by two single reviewers. Doubts and discrepancies regarding surgery results were solved by the authors (MY and XW). The variables between the two groups were also investigated, such as gender, CP diagnosis, and the number of polyps and their size.

### 2.7. Patients Grouping

After surgery, 120 patients were recruited by two investigators according to inclusion and exclusion criteria and assigned to the aspirin group (80 mg aspirin orally administered at 8:00 AM each day) and the BR group (2 g BR orally administered at 8:00 AM each day) [17] using the random number generated by a computer. The intervention was not revealed, and each patient received her or his number using a letter envelope. All result data were analyzed by two statistic experts. The follow-up duration was one year, and the patients would be excluded from the present experiment if he or she wanted to give up the present intervention or severe side effects occurred and needed further treatment.

### 2.8. Inflammatory Analysis

Before the experiment, after one-month surgery and one-year surgery, 10 ml blood was obtained from each patient via venipuncture, and 3 ml blood was used for the measurement of plasma inflammatory cytokines. The levels of TNF-*α* (ab181421), IL-1*β* (ab46052), IL-6(ab46027), and IL-10 (ab46034) in serum were assessed by using ELISA kits from Abcam (Chicago, IL USA) and an automatic blood chemical analyzer (Hitachi, Tokyo, Japan).

The pretreatment neutrophil-to-lymphocyte ratio (NLR) is an independent predictor of prognosis in increased risk of CPs [18]. Immunohistochemical staining for CPs was performed using formalin-fixed, paraffin-embedded CPs tissue sections (4 *μ*m thick) according to a previous report. Sections were then dewaxed in xylene, stained in hematoxylin and eosin (H&E), dehydrated, and mounted with a coverslip [19]. NLR has been widely used as a predictive factor for inflammatory status [20–23] and analyzed in the present work.

### 2.9. Plasma Angiogenin-2 and PKB/Akt Concentrations

Two ml from the previously mentioned blood was used for the measurement of angiogenin-2 and PKB/Akt. Plasma angiogenin-2 (ab99970) and PKB/Akt (ab100662) were measured using the kits from Abcam (Cambridge, MA, USA).

### 2.10. Primary Outcomes

After one-month surgery, primary outcomes were measured, including polyp detection, polyp removal, diagnostic biopsy, and plasma levels of inflammatory cytokines, angiogenin-2, and PKB/Akt. Polyp detection was defined according to ICD-9-CM codes 211.3 and 211.4 on the colonoscopy claim [24]. According to the size classification of CP (≤5 mm, small; 6–9 mm, medium; ≥10 mm, large) [25], more generally, the CP ≥10 mm in diameter was resected [26]. Therefore, patients with large CP were included in the present study after the CP was accurately diagnosed with gastrointestinal endoscopy as Figure S1 shows [27].

### 2.11. Secondary Outcomes

After one-year follow-up, the secondary outcomes were measured, including the recurrence rate of resected CPs, side effects (rapid breathing, seizure; severe nausea, vomiting, and stomach pain; vomit and fever), and plasma levels of angiogenin-2 and PKB/Akt. Liver injury was examined by measuring the related biochemical indices from 5-mL serum, including alanine aminotransferase (ALT), aspartate aminotransferase (AST), alkaline phosphatase (ALP), *γ*-glutamyl transferase (GGT), total bilirubin (TBIL), direct bilirubin (DBIL), total protein (TP), albumin (ALB), and globulin (G). All these parameters were measured by using the kits from Zhejiang ERKN Biotechnology Co. Ltd (Wenzhou, China).

### 2.12. Statistical Analysis

All data were presented as mean values ± standard deviation (SD). The recurrence rate of resected CPs was evaluated using the Kaplan-Meier estimate along with the 95 % confidence interval (CI). *t*-tests, analysis of variance (ANOVA), and chi-square tests were used to compare the variables. The test of Spearman correlation coefficients was used to explore the relationship between the levels of serum bupleurosides and plasma Ang PKB/Akt. All statistical analyses were performed by using SPSS version 20.0 (SPSS Inc., Chicago, IL, USA). *p* < 0.05 was considered statistically significant.

## 3. Results

### 3.1. The Main Components of BR

**Figure 1:**
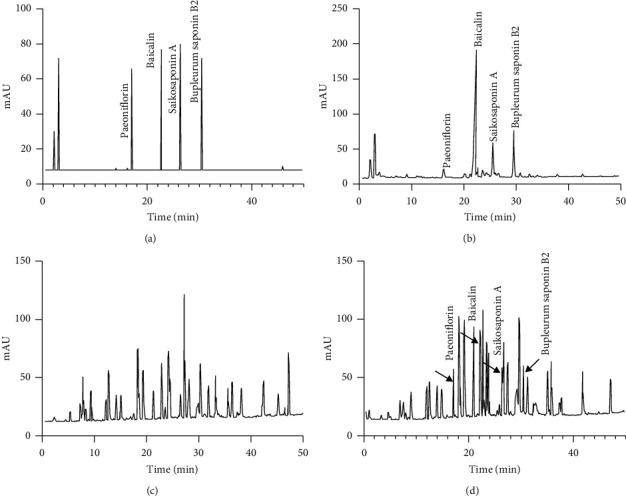
HPLC analysis of bupleurosides levels among different groups. (a) Standard samples. (b) The extracts of BR. (c) Serum level of compounds in the AG group. (d) Serum level of compounds in the BG group. *n* = 55 and 57 for AG and BG groups, respectively.

Comparing with the standards (Figure 1(a)), the main components of BR are paeoniflorin, baicalin, saikosaponin A, and bupleurum saponin B2 (Figure 1(b)). HPLC analysis showed that there were no such compounds in the serum from the AG group (Figure 1(c)), and the main compounds of BR could be found in the serum from the BG group (Figure 1(d) and Table S1).

### 3.2. Baseline Characters

**Figure 2:**
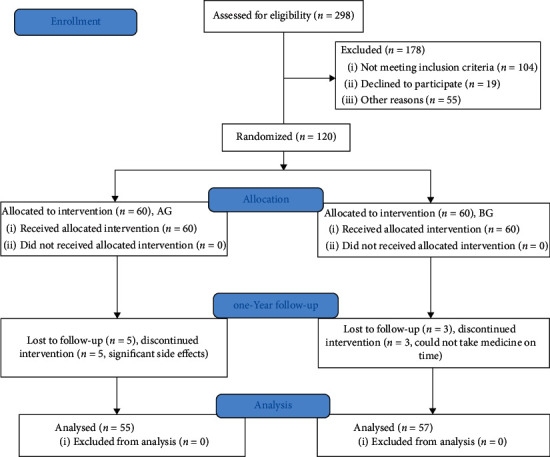
CONSORT flowchart of enrollment, treatment, and follow-up.

The complete polyp resection was achieved in all the polyps and marginal biopsies investigation demonstrated that no residual polyp tissue was found. After one-month surgery, no one withdrew from the present study. There were stable results and no significant side effects. After one-year follow-up, five patients were removed from the AG group because of significant side effects, and three patients were removed from the BG group because they could not take the medicine on time for more than five days. Thus, 55 and 57 patients finished the present study from the AG and BG groups (Figure 2), respectively. The number of changes would not affect the final statistical results. The statistical difference was insignificant for median age, gender distribution, the duration of CPs, polyp characteristics, CP histology, and resecting technique between the two groups (Table 1, *p* < 0.05).

The values of NLR in most samples were similar between the two groups and the statistical difference was insignificant (Figure S2, *p* > 0.05). The neoplastic polyps (tubular adenoma, tubulovillous adenoma, and adenocarcinoma) had higher inflammatory status than the nonneoplastic polyps (serrated and hyperplastic, Table 1) (Figure S2, *p* < 0.05).

### 3.3. Primary Outcomes

**Figure 3:**
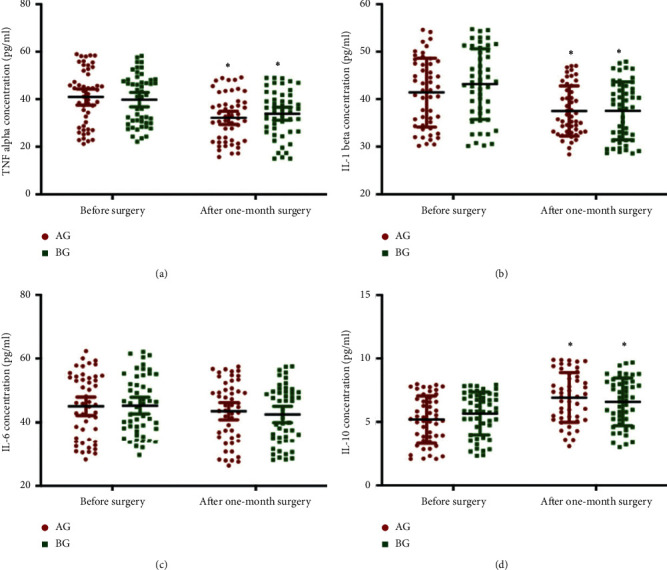
Plasma levels of inflammatory cytokines in primary outcomes after one-month surgery. (a) TNF-*α*. (b) IL-1*β*. (c) IL-6. (d) IL-10. *n* = 55 and 57 for AG and BG groups, respectively. ^*∗*^*p* < 0.05 vs. before surgery.

**Figure 4:**
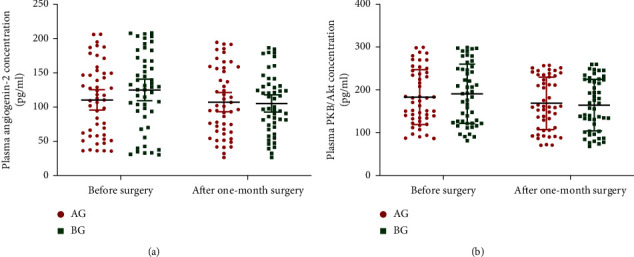
Plasma levels of angiogenic factors in primary outcomes after one-month surgery. (a) Angiogenin-2. (b) PKB/Akt. *n* = 55 and 57 for AG and BG groups, respectively.

ELISA analysis showed that the statistical difference for the plasma levels of TNF-*α* (Figure 3(a)), IL-1*β* (Figure 3(b)), IL-6 (Figure 3(c)), and IL-10 (Figure 3(d)) was insignificant before surgery and after one-month surgery between the two groups (Figure 3, *p* > 0.05). Similarly, ELISA analysis showed that the statistical difference for the plasma levels of angiogenin-2 (Figure 4(a)) and PKB/Akt (Figure 4(b)) was insignificant before surgery and after one-month surgery between two groups (Figure 4, *p* > 0.05).

### 3.4. Secondary Outcomes

The normal side effects included rapid breathing and seizure; severe nausea, vomiting, and stomach pain; vomit and fever in both groups. The side effects (rapid breathing and seizure; severe nausea and stomach pain) in the AG group was higher than that in the BG group (Table 2, *p* < 0.05). The results suggest BR treatment had fewer side effects than aspirin treatment.

**Figure 5:**
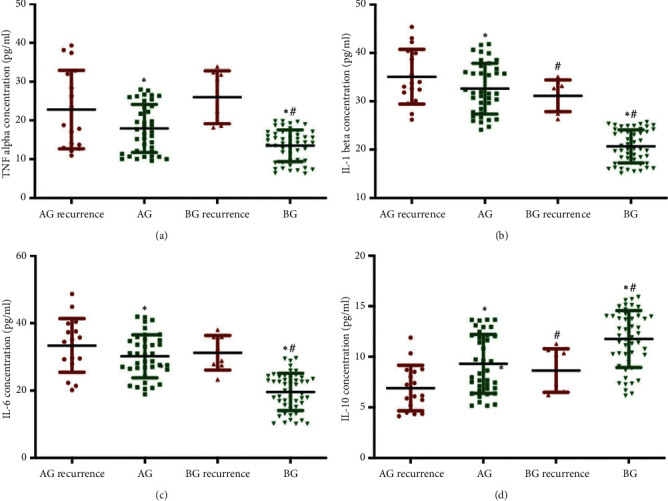
Plasma levels of inflammatory cytokines in secondary outcomes after one-year follow-up. (a) TNF-*α*. (b) IL-1*β*. (c) IL-6. (d) IL-10. *n* = 55 and 57 for AG and BG groups, respectively. Symbols *∗* and # stand for the significant difference between the two groups. *∗p* < 0.05 vs. the recurrence of resected CP group and #*p* < 0.05 vs. the AG group.

ELISA analysis showed that the statistical difference for the plasma levels of TNF-*α* (Figure 5(a)), IL-1*β* (Figure 5(b)), IL-6 (Figure 5(c)), and IL-10 (Figure 5(d)) was significant after one-year surgery between the two groups (Figure 5, *p* < 0.05). Meanwhile, the plasma levels of TNF-*α* (Figure 5(a)), IL-1*β* (Figure 5(b)), and IL-6(Figure 5(c)) in the group with the recurrence of resected CP were higher than those in the group without recurrence, whereas the level of IL-10 (Figure 5(d)) in the group with the recurrence of resected CP was lower than that in the group without recurrence (Figure 5, *p* < 0.05). The results suggest that inflammatory cytokines are important factors to affect the recurrence of resected CP.

**Figure 6:**
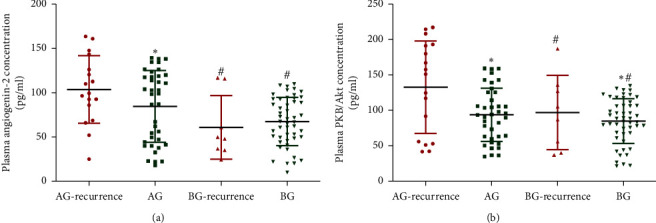
Plasma levels of angiogenic factors in secondary outcomes after one-year follow-up. (a) Angiogenin-2. (b) PKB/Akt. *n* = 55 and 57 for AG and BG groups, respectively. Symbols *∗* and # stand for the significant difference between the two groups. ^*∗*^*p* < 0.05 vs. the recurrence of resected CP group and ^#^*p* < 0.05 vs. the AG group.

Similarly, ELISA analysis showed that the statistical difference for the plasma levels of angiogenin-2 (Figure 6(a)) and PKB/Akt (Figure 6(b)) was significant before surgery and after one-year surgery between the two groups (Figure 6, *p* < 0.05). The results suggest BR treatment reduced inflammatory responses and plasma levels of angiogenin-2 and PKB/Akt.

The recurrence of resected CPs was investigated at follow-up colonoscopy at the exact polyp site or in the colonic segment having index polypectomy. The recurrence rate of resected CPs in the AG group was higher than that in the BG group (Table 3, *p* < 0.05). The results suggest BR treatment reduced resected CP recurrence rate better than aspirin intervention.

### 3.5. Serum Paeoniflorin, Baicalin, Saikosaponin A, and Bupleurum Saponin B2 Had a Strong Negative Relationship with the Plasma Levels of Ang PKB/Akt

**Figure 7:**
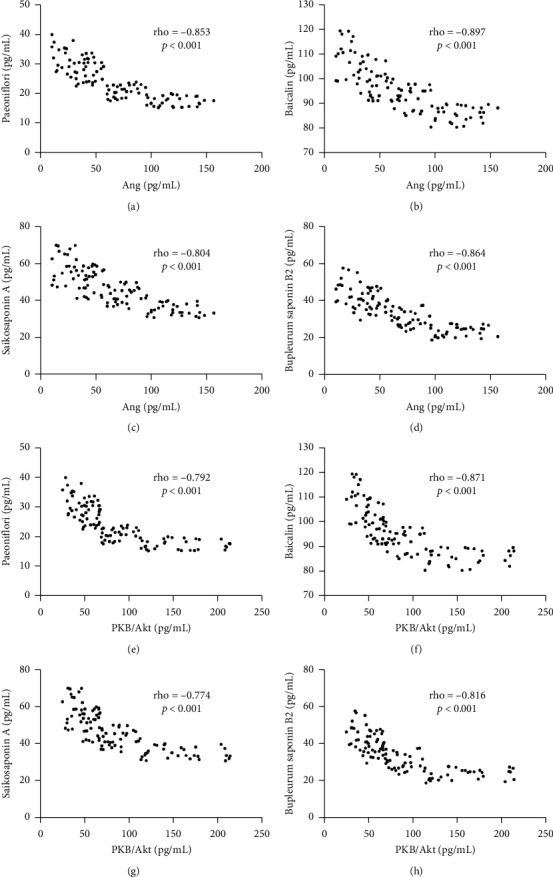
The relationship between the levels of serum bupleurosides and plasma Ang PKB/Akt. (a) Paeoniflorin and Ang. (b) Baicalin and Ang. (c) Saikosaponin A and Ang. (d) Bupleurum saponin B2 and Ang. (e) Paeoniflorin and PKB/Akt. (f) Baicalin and PKB/Akt. (g) Saikosaponin A and PKB/Akt. (h) Bupleurum saponin B2 and PKB/Akt. The serum levels of compounds were provided in BG group (Table S1) There was a strong negative relationship if rho <−0.50.

The test of Spearman correlation coefficients showed that serum levels of paeoniflorin (Figure 7(a)), baicalin (Figure 7(b)), saikosaponin A (Figure 7(c)), and bupleurum saponin B2 (Figure 7(d)) were increasing with the reduction in the levels of plasma levels of Ang. Serum paeoniflorin, baicalin, saikosaponin A, and bupleurum saponin B2 had a strong negative relationship with the plasma levels of Ang (*p* < 0.001). Similarly, serum levels of paeoniflorin (Figure 7(e)), baicalin (Figure 7(f)), saikosaponin A (Figure 7(g)), and bupleurum saponin B2 (Figure 7(h)) were increasing with the reduction in the levels of plasma levels of PKB/Akt. Serum paeoniflorin, baicalin, saikosaponin A, and bupleurum saponin B2 had a strong negative relationship with the plasma levels of PKB/Akt (*p* < 0.001).

### 3.6. BR Treatment Did Not Cause Liver Injury according to Serum Biochemical Analysis

Serum biochemical analysis showed that the all the parameters were similar between the two groups, including ALT, AST, ALP, GGT, TBIL, DBIL, TP, ALB, and G (Table 4, *p* > 0.05). The results suggest that BR treatment will not cause liver injury according to serum biochemical analysis.

## 4. Discussion

CP is relatively easier to cure than colorectal cancer (CRC), but if not treated in time, colorectal polyps possibly develop into colorectal cancer [28]. CRC is the third most common cancer worldwide and the fourth most common cause of mortality [29]. Earlier detection of CP will improve survival rate of the patients [30]. Comparatively, it will take a long time and the cancerous rate of colonic polyp is low, and colorectal polyp is associated with greater morbidity than colonic polyp [31, 32].

In our cohort of patients, the effects of BR on the recurrence rate of resected CPs were explored. Overall, our study concluded that BR was effective and a safe therapy for the patients with polyps  > 10 mm undergoing CP resection. BR effectively prevented the recurrence rate of resected CPs by reducing the plasma levels of proinflammatory cytokines TNF-*α* (Figure 5(a)), IL-1*β* (Figure 5(b)), and IL-6(Figure 5(c)) and increasing the level of anti-inflammatory cytokine IL-10 (Figure 5(d)). More importantly, BR significantly reduced the plasma levels of vascular angiogenic factors angiogenin-2 [33, 34] and PKB/Akt, which play important roles in the growth and progression of polyps, and colorectal carcinogenesis. The inflammatory cells play an important role in inflammatory infiltrate, and NLR values were analyzed in primary biopsies (Figure S2). However, the new biopsies were seldom found and the CP size was small, and the NLR data were not compared between the two groups after one-year treatment.

BR is a very popular herbal medicine in China, and its anti-inflammatory properties have been reported [35, 36]. Our results also showed the similar results by improving anti-inflammatory activities in the CP patients. Inflammatory cytokines are important factors to affect the recurrence of resected CP. The inhibitory function of BR on the level of PKB/Akt has been reported in human umbilical vein endothelial cells (HUVECs) [37]. However, the potential therapeutic effects of BR on various cancers have also been widely reported [38–42]. However, the effects of BR on polyps, especially CP, have seldom been reported. There was still no report for the effects of BR on the expression of angiogenin-2 before the present work although angiogenin-2 plays a crucial role in the progression and risk of polyps [43] and tumorigenesis [44–47]. Correlation test showed that the main ingredients paeoniflorin, baicalin, saikosaponin A, and bupleurum saponin B2 had a strong negative relationship with the plasma levels of Ang PKB/Akt (Figure 7). Thus, BR may exert its function via these main compounds.

More importantly, BR has a few side effects as natural products and causes fewer side effects or little liver injury when compared with aspirin therapy (Tables 2 and 4). BR has been reported to reduce 6-hydroxydopamine- (6-OHDA-) induced cytotoxicity in vitro and in vivo [48]. BR is often used with clozapine in schizophrenia patients to reduce its side effects and improve therapeutic efficacy [49]. All these results suggest that BR is a potential drug for CP therapy with high safety.

## 5. Limitations

There were some limitations to the present study: all polyp diameters > 10 mm were included in the work, including both polypoid and flat. However, more than 30% of the polyps were nonpolypoid and therefore clinically very relevant. The recurrence of resected CPs in the larger polyps was significantly more than in the smaller ones, and they were also still endoscopically treatable. The follow-up and surveillance procedures performed were conducted at our hospital, which made the final data more meaningful. The experiments were performed by many different gastroenterologists, who had different techniques in the surgery. The present data showed the majority of gastroenterology practices. The long-term effects of BR on the recurrence of resected CPs are still needed to be explored in the future. The classification of colonic types is complex (Figure S3) and cannot be applied to our small population sample in this study. In the present work, the colonic polyps were schematically divided into two major polyp types, neoplastic polyps (tubular adenoma, tubulovillous adenoma, and adenocarcinoma) and nonneoplastic polyps (serrated and hyperplastic, Table 1). Serrated polyp is similar in morphology to the hyperplastic polyp, but the former has a malignant potential. Only a few types of colonic polyps were found with a small number in the present work. Statistical analysis cannot be performed for most colonic polyp types. These important issues should be addressed in a larger population sample in the future work.

## 6. Conclusion

In conclusion, the effects of BR on the recurrence of resected CP were explored here. We show that BR is a feasible and curative process, which may help reduce the recurrence of resected CP in CP patients who underwent CP by reducing the plasma levels of inflammatory cytokines and vascular angiogenic factors angiogenin-2 and PKB/Akt and increasing the level of anti-inflammatory cytokines. BR might be used as a potential drug in the prevention of the progression and risk of CPs via its main ingredients paeoniflorin, baicalin, saikosaponin A, and bupleurum saponin B2.

## Figures and Tables

**Table 1:** Baseline characters of two groups.

Parameters	AG, *n* = 55	BG, *n* = 57	*t* or *χ*^2^ values	*p*
Median age, years (95% CI)	49.1 (40.2–85.3)	51.2 (42.4–88.6)	0.654	0.279
Median duration of disease, years (95% CI)	6.3 (0.5–15.6)	6.6 (0.5–12.9)	0.563	0.426
Gender, male cases (%)	37 (67.3)	40 (70.2)	0.110	0.740
*Polyp characteristics*				
Median size of polyp, mm (95% CI)	16.4 (11.2–26.1)	15.1 (12.4–25.7)	0.758	0.134
Ascending colon	20 (36.4)	19 (33.3)	0.827	0.935
Transverse colon	8 (14.5)	10 (17.5)		
Descending colon	4 (7.3)	6 (10.5)		
Sigmoid colon	13 (23.6)	11 (19.3)		
Rectum	10 (18.2)	11 (19.3)		

*Histology*, *n (%)*				
Tubular adenoma	31 (56.4)	32 (56.1)	1.638	0.802
Tubulovillous adenoma	6 (10.9)	5 (8.8)		
Adenocarcinoma	1 (1.8)	3 (5.3)		
Serrated	11 (20)	13 (22.8)		
Hyperplastic	6 (10.9)	4 (7)		

*Resectioning technique*, *n (%)*				
En bloc	37 (67.3)	35 (61.4)	0.420	0.517
Piecemeal	18 (32.7)	22 (38.6)		

*t*-test for continuous data and Chi-square for count number. The statistical difference was significant if *p* < 0.05.

**Table 2:** Side effects between the two groups.

Side effects, *n* (%)	AG, *n* = 55	BG, *n* = 57	*χ* ^2^ values	*p*
Rapid breathing	11 (20)	1 (1.8)	9.741	0.002
Seizure	6 (10.9)	4 (7.3)	0.153	0.696
Severe nausea	12 (21.8)	2 (3.6)	8.579	0.003
Vomiting	13 (23.6)	10 (18.2)	0.637	0.425
Stomach pain	18 (32.7)	3 (5.5)	13.859	0.000
Vomit	4 (7.3)	2 (3.6)	0.216	0.642
Fever	3 (5.5)	1 (1.8)	0.298	0.585

Chi-square for count number. The statistical difference was significant if *p* < 0.05.

**Table 3:** Recurrence of CPs between the two groups, *n* (%).

Types	AG, *n* = 55	BG, *n* = 57	*χ* ^2^ values	*p*
1 recurrence	11 (20)	6 (20)	4.597	0.032
2 recurrences	5 (9.1)	1 (9.1)		
3 recurrences	1 (1.8)	1 (1.8)		

Chi-square for count number. The statistical difference was significant if *p* < 0.05.

**Table 4:** Biochemical analysis of liver injury.

Parameters	Normal range	AG, *n* = 55	BG, *n* = 57	*t* values	*p*
ALT	0–45 IU/dL	26.8 ± 11.2	28.1 ± 12.4	0.763	0.365
AST	0–40 IU/dL	19.6 ± 9.3	20.6 ± 10.9	0.096	0.898
ALP	0–500 mg/dL	154.8 ± 63.9	156.4 ± 64.2	0.023	0.965
GGT	10–60 mg/dL	36.7 ± 15.7	37.2 ± 17	0.136	0.872
TBIL	3.4–21.7 mg/dL	10.4 ± 4.7	11.7 ± 5.4	0.890	0.138
DBIL	0.4–6.8 mg/dL	3.8 ± 3.1	4.1 ± 3.5	0.769	0.212
TP	60–85 mg/dL	73.5 ± 29.7	74.3 ± 29.8	0.056	0.831
ALB	35–55 mg/dL	42.9 ± 18.3	44.4 ± 18.5	0.964	0.128
G	15–40 mg/dL	30.6 ± 13.1	31.8 ± 14.4	0.752	0.104

*t*-test was used to compare the statistical difference between the two groups. Alanine aminotransferase (ALT), aspartate aminotransferase (AST), alkaline phosphatase (ALP), *γ*-glutamyl transferase (GGT), total bilirubin (TBIL), direct bilirubin (DBIL), total protein (TP), albumin (ALB), and globulin (G). The statistical difference was significant if *p* < 0.05.

## Data Availability

The data are available from the corresponding author upon reasonable request.

## Supplementary Materials

Supplementary Materials Figure S1: colorectal polyps were diagnosed with gastrointestinal endoscopy. Figure S2: neutrophil-to-lymphocyte ratio (NLR) between two groups. *n* = 38 and 17 for neoplastic and nonneoplastic samples in the AG group, and *n* = 40 and 17 for neoplastic and nonneoplastic samples in the BG group. The statistical difference was significant if *p* < 0.05 vs. the neoplastic group. Figure S3: classification of colonic polyps. Table S1: the concentrations of Ang and PKB/Akt, and the serum levels of compounds from BR in the BG group.
