# Supplementary material for: Bupleuri Radix Prevents the Recurrences of Resected Colonic Polyps by Affecting Angiogenin-2-Induced Protein Kinase B/Akt Signaling
Source: J Oncol. 2020 Oct 28;2020:3531652. doi: 10.1155/2020/3531652 (PMC7657685; doi:10.1155/2020/3531652)
Supplement: Supplementary Materials — Figure S1: colorectal polyps were diagnosed with gastrointestinal endoscopy. Figure S2: neutrophil-to-lymphocyte ratio (NLR) between two groups. n = 38 and 17 for neoplastic and nonneoplastic samples in the AG group, and n = 40 and 17 for neoplastic and nonneoplastic samples in the BG group. The statistical difference was significant if p < 0.05 vs. the neoplastic group. Figure S3: classification of colonic polyps. Table S1: the concentrations of Ang and PKB/Akt, and the serum levels of compounds from BR in the BG group. [file 3531652.f1.doc]

***Bupleuri Radix* prevents the recurrences of resected colonic polyps by affecting angiogenin-2-induced protein kinase B/Akt signaling**

Qiang Gao^1,#^, Guihong Yu^2,#^, Minghui Yu^3^, Xinping Wang^4,*^

^1^Department of Spleen-stomach disease, Yantai Hospital of Traditional Chinese Medicine, Yantai 264002, China

^2^Department of Integrated TCM & Western Medicine, Yantai Qishan Hospital, Yantai 264000, China

^3^Department of Clinical Laboratory, Yantai Wanhua Hospital, Yantai 260000, China

^4^Department of Clinical Laboratory, Yantai Hospital of Traditional Chinese Medicine, Yantai, 264002, China

Figure S1


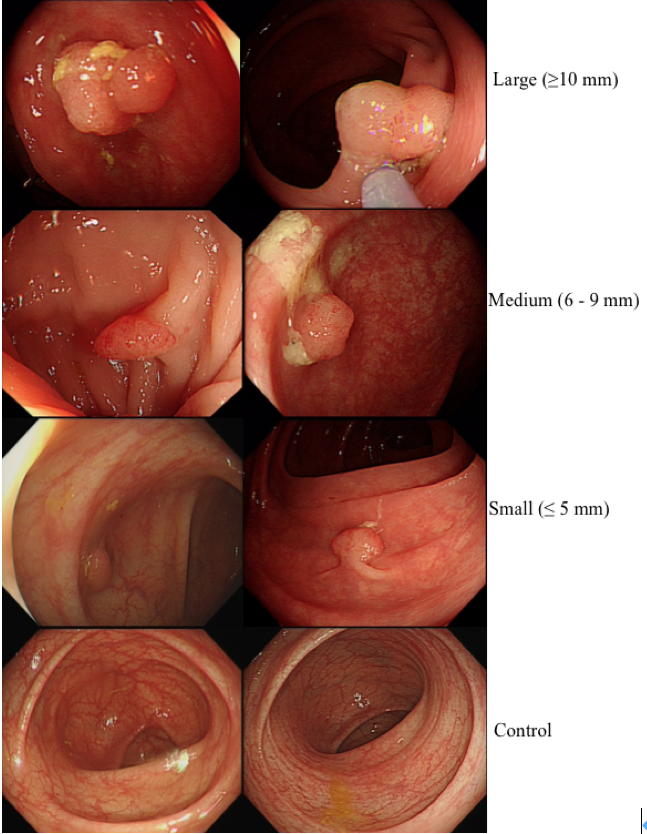


Figure S1. Colorectal polyps were diagnosed with gastrointestinal endoscopy.

Figure S2


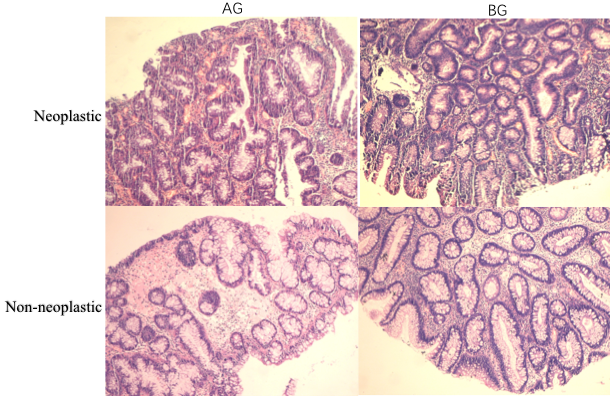


Figure S2: Neutrophil-to-lymphocyte ratio (NLR) between two groups. n=38 and 17 for neoplastic and non-neoplastic samples in the AG group and n=40 and 17 for neoplastic and non-neoplastic samples in the BG group. The statistical difference was significant if p < 0.05 vs the neoplastic group.

Figure S3

Figure S3. Classification of colonic polyps.

Table S1. The concentrations of Ang and PKB/Akt, and the serum levels of compounds from BR in the BG group.

| Ang (pg/mL) | PKB/Akt (pg/mL) | Paeniflori (pg/mL) | Baiclin  (pg/mL) | Saikosaponin a (pg/mL) | Bupleurum saponin b2 (pg/mL) |
| --- | --- | --- | --- | --- | --- |
| 156.6 | 214.1 | 17.6 | 88.1 | 33.2 | 20.5 |
| 148.2 | 213.2 | 17.7 | 89.5 | 31.9 | 26.6 |
| 143.9 | 210.1 | 16.4 | 86.3 | 33.5 | 24.6 |
| 143.3 | 209.1 | 16.7 | 81.9 | 30.7 | 27.2 |
| 141.7 | 208.4 | 15.5 | 88.1 | 37.3 | 24.9 |
| 141.6 | 204.1 | 19.1 | 84.3 | 39.6 | 19.4 |
| 140.6 | 179.3 | 19.0 | 86.3 | 31.4 | 22.3 |
| 134.3 | 177.9 | 15.6 | 88.2 | 33.1 | 24.4 |
| 132.0 | 175.5 | 19.2 | 84.2 | 38.1 | 25.5 |
| 127.9 | 173.8 | 15.3 | 83.5 | 33.5 | 20.8 |
| 127.6 | 169.1 | 15.4 | 89.0 | 31.7 | 24.7 |
| 125.8 | 164.5 | 18.2 | 80.7 | 33.5 | 24.7 |
| 123.5 | 162.2 | 19.5 | 89.2 | 36.8 | 24.4 |
| 120.1 | 159.2 | 15.3 | 89.5 | 37.8 | 25.4 |
| 119.5 | 155.5 | 15.6 | 80.3 | 33.4 | 25.4 |
| 119.5 | 148.5 | 19.6 | 85.0 | 35.0 | 27.1 |
| 118.2 | 143.8 | 19.9 | 85.7 | 32.1 | 20.0 |
| 115.1 | 140.6 | 18.2 | 81.7 | 39.9 | 27.5 |
| 113.6 | 139.9 | 17.7 | 82.3 | 39.4 | 27.6 |
| 110.7 | 139.2 | 19.3 | 89.4 | 37.0 | 23.0 |
| 110.0 | 131.6 | 16.8 | 89.7 | 39.1 | 21.9 |
| 107.8 | 129.0 | 15.8 | 86.5 | 39.5 | 20.2 |
| 106.7 | 121.9 | 15.2 | 88.2 | 35.9 | 21.2 |
| 102.9 | 120.7 | 18.3 | 86.5 | 34.9 | 23.7 |
| 102.5 | 119.6 | 15.6 | 88.0 | 30.8 | 19.8 |
| 100.2 | 118.8 | 16.7 | 83.8 | 34.7 | 21.0 |
| 99.9 | 118.1 | 16.6 | 83.0 | 33.8 | 20.4 |
| 97.0 | 114.7 | 18.6 | 88.9 | 32.9 | 18.6 |
| 96.2 | 113.7 | 16.0 | 80.3 | 31.3 | 26.7 |
| 96.0 | 112.8 | 19.5 | 95.3 | 42.7 | 25.1 |
| 95.0 | 110.8 | 22.0 | 97.5 | 41.4 | 31.6 |
| 90.8 | 108.2 | 23.0 | 95.0 | 41.0 | 27.9 |
| 88.9 | 107.6 | 20.1 | 95.0 | 46.4 | 37.4 |
| 87.7 | 105.1 | 21.6 | 86.8 | 47.2 | 37.3 |
| 86.6 | 98.3 | 23.8 | 85.7 | 49.7 | 27.5 |
| 84.7 | 97.6 | 23.0 | 91.9 | 45.5 | 29.6 |
| 82.0 | 96.6 | 20.6 | 97.7 | 44.5 | 33.0 |
| 80.4 | 93.3 | 22.8 | 87.2 | 45.7 | 24.9 |
| 79.8 | 91.2 | 23.6 | 86.8 | 35.6 | 29.6 |
| 78.0 | 91.1 | 20.7 | 94.5 | 38.3 | 34.0 |
| 77.9 | 90.6 | 20.6 | 96.1 | 42.3 | 24.3 |
| 77.2 | 89.1 | 18.5 | 95.0 | 49.9 | 28.2 |
| 76.0 | 88.7 | 21.5 | 97.7 | 49.0 | 27.6 |
| 73.6 | 84.3 | 21.4 | 85.0 | 39.0 | 23.4 |
| 73.5 | 83.8 | 18.4 | 91.3 | 45.3 | 26.7 |
| 72.2 | 82.3 | 22.8 | 86.0 | 50.0 | 34.0 |
| 69.6 | 81.9 | 17.8 | 95.3 | 40.8 | 35.2 |
| 69.5 | 80.7 | 22.3 | 95.3 | 37.8 | 25.4 |
| 68.4 | 79.0 | 20.4 | 93.0 | 40.6 | 26.7 |
| 67.8 | 77.8 | 18.3 | 91.0 | 39.3 | 36.1 |
| 67.3 | 76.2 | 20.2 | 92.5 | 40.9 | 26.0 |
| 67.0 | 74.2 | 20.4 | 95.9 | 45.6 | 30.8 |
| 66.3 | 72.8 | 18.8 | 93.1 | 40.9 | 26.9 |
| 65.9 | 71.0 | 22.9 | 91.5 | 36.8 | 30.0 |
| 63.1 | 71.0 | 17.5 | 87.8 | 43.8 | 30.9 |
| 61.8 | 69.6 | 21.2 | 99.8 | 47.3 | 36.9 |
| 61.0 | 69.5 | 18.0 | 90.8 | 48.1 | 29.1 |
| 60.3 | 69.4 | 22.6 | 98.3 | 38.7 | 34.3 |
| 60.3 | 68.7 | 19.8 | 91.3 | 36.9 | 31.8 |
| 57.1 | 67.5 | 29.1 | 96.3 | 56.6 | 40.6 |
| 56.9 | 66.7 | 29.1 | 100.3 | 48.6 | 40.2 |
| 55.9 | 66.4 | 24.8 | 99.0 | 56.1 | 39.8 |
| 55.2 | 66.3 | 30.5 | 107.2 | 41.7 | 33.6 |
| 54.8 | 66.3 | 28.4 | 101.8 | 42.9 | 36.6 |
| 50.9 | 65.9 | 27.4 | 101.1 | 58.6 | 46.7 |
| 49.6 | 65.8 | 24.1 | 93.1 | 54.0 | 38.0 |
| 49.1 | 65.8 | 30.4 | 93.0 | 59.3 | 38.8 |
| 48.2 | 64.9 | 32.3 | 91.1 | 44.2 | 45.3 |
| 47.9 | 63.6 | 26.2 | 101.2 | 55.4 | 37.2 |
| 47.2 | 63.2 | 23.7 | 101.0 | 59.8 | 47.6 |
| 47.0 | 61.9 | 22.8 | 97.2 | 53.0 | 35.8 |
| 46.5 | 60.9 | 33.7 | 107.8 | 58.7 | 32.0 |
| 43.9 | 60.2 | 24.1 | 91.0 | 53.0 | 42.2 |
| 43.3 | 59.1 | 31.7 | 92.2 | 57.7 | 41.0 |
| 43.1 | 59.0 | 30.5 | 97.0 | 40.8 | 47.0 |
| 42.5 | 57.2 | 31.8 | 102.8 | 48.2 | 35.5 |
| 41.6 | 56.7 | 23.9 | 93.4 | 51.6 | 45.2 |
| 41.4 | 54.9 | 33.6 | 93.0 | 41.6 | 32.5 |
| 40.9 | 54.4 | 29.0 | 99.5 | 53.6 | 36.4 |
| 40.7 | 52.4 | 26.0 | 94.6 | 46.8 | 46.7 |
| 39.8 | 52.4 | 31.6 | 91.2 | 51.2 | 32.7 |
| 39.4 | 51.6 | 30.3 | 109.7 | 42.1 | 43.9 |
| 39.2 | 51.2 | 28.1 | 109.5 | 46.9 | 47.2 |
| 38.7 | 50.4 | 23.7 | 98.8 | 58.7 | 38.4 |
| 37.7 | 49.5 | 33.0 | 104.0 | 56.1 | 35.1 |
| 34.9 | 49.5 | 30.5 | 106.8 | 46.7 | 40.1 |
| 34.1 | 48.5 | 23.2 | 109.1 | 53.6 | 43.8 |
| 32.8 | 48.1 | 27.0 | 94.1 | 41.2 | 40.9 |
| 32.4 | 47.9 | 22.5 | 106.1 | 56.2 | 29.4 |
| 32.3 | 47.4 | 25.0 | 103.6 | 62.2 | 50.2 |
| 31.3 | 46.5 | 27.3 | 100.2 | 69.9 | 37.5 |
| 30.6 | 46.5 | 30.2 | 102.7 | 58.2 | 40.8 |
| 29.6 | 45.9 | 37.9 | 97.9 | 51.8 | 55.2 |
| 27.6 | 44.5 | 25.5 | 110.7 | 55.5 | 35.7 |
| 26.5 | 43.6 | 31.3 | 101.4 | 68.0 | 33.4 |
| 25.9 | 40.4 | 29.1 | 117.1 | 58.6 | 36.4 |
| 24.4 | 38.2 | 26.6 | 115.1 | 64.9 | 42.0 |
| 23.5 | 37.7 | 35.2 | 111.4 | 58.5 | 46.2 |
| 22.6 | 36.0 | 33.7 | 106.6 | 47.9 | 41.8 |
| 21.7 | 35.9 | 35.4 | 119.2 | 65.1 | 56.6 |
| 19.5 | 35.3 | 28.8 | 99.6 | 54.8 | 38.2 |
| 16.7 | 34.6 | 34.8 | 110.6 | 66.7 | 57.5 |
| 15.8 | 33.4 | 29.6 | 118.1 | 69.6 | 48.2 |
| 15.1 | 31.4 | 27.8 | 112.1 | 47.4 | 52.1 |
| 14.2 | 31.2 | 27.4 | 119.4 | 70.0 | 48.6 |
| 12.3 | 30.5 | 37.4 | 110.1 | 53.3 | 48.5 |
| 11.8 | 30.1 | 32.0 | 99.0 | 51.1 | 39.9 |
| 10.4 | 28.2 | 39.9 | 99.2 | 48.3 | 39.4 |
| 10.4 | 24.9 | 35.8 | 109.1 | 62.6 | 46.3 |
